# Response to Letter: "Use of Electroanatomic Mapping Systems beyond Electrophysiologic Studies"

**DOI:** 10.1016/s0972-6292(16)30653-2

**Published:** 2013-08-01

**Authors:** Alejandro Velasco, Victor Manuel Velasco, Fernando Rosas, Cihan Cevik, Carlos A Morillo

**Affiliations:** 1 Internal Medicine Department. Texas Tech University Health Sciences Center, Lubbock, Texas. USA; 2 Electrophysiology and Cardiac Stimulation Service. Fundacion Clinica Shaio, Bogota. Colombia; 3 Cardiology Department, Texas Heart Institute at St.Luke's Episcopal Hospital, Baylor College of Medicine, Houston, Texas. USA; 4 Arrhythmia Services, Hamilton Health Sciences. Mc Master University, Hamilton, Ontario. Canada

**Keywords:** Electroanatomic Mapping

We appreciated hearing from Dr. Cay regarding our recent published case report that demonstrates the implantation of a permanent pacemaker in a pregnant patient with Chagas disease using the NavX® electroanatomic mapping system to reduce the exposure to ionizing radiation [1]. As Dr. Cay mentions the radiation dose necessary to produce harm in a 31-week fetus is probably greater than the dose used with fluoroscopy for implanting a cardiac device. However it is known that pregnant patients that undergo procedures involving radiation have a high perception of teratogenic risk [2] and may develop increased levels of anxiety during and after the procedure. Also, the damage produced by radiation is difficult to quantify and involves loss of tissue function or deterministic effects, which are dose dependent, and stochastic effects, which are random and not dose dependent [3]. As the American College of Obstetrics and Gynecology recommends, during pregnancy the use of imaging procedures not associated with ionizing radiation should be used when appropriate [4], and we have successfully demonstrated a method to implant an atrial and a ventricular pacing lead with minimal radiation exposure in a pregnant patient

Regarding the position of the ventricular lead documented by the chest X-Ray we agree with Dr. Cay that the lead is not in the right ventricle outflow tract, the lead is located in the mid-septal area of the right ventricle.
